# Association between IFNGR1 gene polymorphisms and tuberculosis susceptibility: A meta-analysis

**DOI:** 10.3389/fpubh.2022.976221

**Published:** 2022-09-06

**Authors:** Liwei Cheng, Fan Zhang, Ying Wang, Jing Chen, Xiaoping Yuan

**Affiliations:** ^1^Office of Academic Research, Renmin Hospital of Wuhan University, Wuhan, China; ^2^Department of Neurology, Renmin Hospital of Wuhan University, Wuhan, China; ^3^Department of Psychiatry, Renmin Hospital of Wuhan University, Wuhan, China; ^4^Outpatient Department, Renmin Hospital of Wuhan University, Wuhan, China

**Keywords:** tuberculosis, interferon gamma receptor 1, polymorphism, susceptibility, metaanalysis

## Abstract

The association of IFN-γ receptor 1 (IFNGR1) gene polymorphisms with tuberculosis (TB) susceptibility has not been systematically studied. We therefore conducted a meta-analysis to assess their association. Literature search was conducted in PubMed, EMBASE, Web of Science, and the Cochrane Library. Odds ratio (OR) and 95% confidence interval (CI) was pooled by the random-effect model. Statistical analyses were performed using STATA 12.0 software. Fourteen studies involved 7,699 TB cases and 8,289 controls were included in this meta-analysis. A significant association was found between the IFNGR1 rs2234711 polymorphism and TB susceptibility among Africans in dominant model (OR = 1.24, 95%CI:1.01–1.52), and among Asians in allele model (OR = 0.89, 95%CI: 0.79–0.99), homozygote model (OR = 0.82, 95%CI: 0.70–0.98) and additive model (OR = 0.90, 95%CI: 0.83–0.97). In addition, a significant association was observed between the IFNGR1 rs7749390 polymorphism and TB susceptibility among Africans in allele model (OR = 0.89, 95%CI: 0.82–0.98). No significant association was found between the IFNGR1 rs1327474 polymorphism and TB susceptibility. In summary, IFNGR1 rs2234711 polymorphism was associated with increased TB susceptibility in Africans and decreased TB susceptibility in Asians, while IFNGR1 rs7749390 polymorphism was associated with decreased TB susceptibility in Africans.

## Introduction

Tuberculosis (TB), a chronic infectious disease caused by the bacterium mycobacterium tuberculosis (M.TB), affects several organs, but mostly attacks the lungs (1). According to the World Health Organization, TB is associated with an estimation of 10 million incident cases (2), among which 8.7 million cases are from 30 high-burden countries (3), and causes approximately 1.3 million deaths worldwide in 2018 (2). Over the past decade, the incidence of drug-resistant TB has continued to increase. Globally, 4.6% of TB cases are multidrug resistant, and in some areas, this proportion exceeds 25% (4). Therefore, TB remains a major public health concern worldwide, especially in developing countries.

Many studies have demonstrated that TB susceptibility is partly determined by the host genetic background (5–7). In support of this, differences in the rates of TB occurrence among ethnicities and families indicate a genetic predisposition to TB susceptibility (8). Interferon-gamma (IFN-γ) is well–known to play a critical role in the activation of macrophages and dendritic cells and influences the innate immune response to certain contagious agents, including M.TB (9). The interferon-gamma receptor 1 (IFNGR1) gene encodes the ligand-binding chain (alpha) of the IFN-γ receptor, which is located on chromosome 6q23.3 (10). After binding to IFN-γ, IFNGR1 could regulate the cytokine response in Natural killer and T cells to exert their microbicidal functions (11, 12). Animal and *in vitro* studies have demonstrated that IFNGR1 plays a pivotal role in the progression of TB (13, 14).

Previous studies have investigated the association between genetic variants of IFNGR1 and TB susceptibility based on the hypothesis that defects or variations in IFNGR1 may result in the increased susceptibility or accelerated progression of diverse diseases, such as inflammatory and virus-associated disorders (15, 16). Three potentially functional single nucleotide polymorphisms (SNPs) of the IFNGR1 gene (rs2234711, rs1327474, and rs7749390) have been investigated to explore their impact on TB susceptibility by several studies (17–30). Nonetheless, the results did not provide consistently significant associations between these SNPs possibly due to the use of sample sizes that did not allow the detection of small genetic effects. Therefore, this meta-analysis was conducted to detect the association of the IFNGR1 rs2234711, rs1327474, rs7749390 polymorphisms with TB susceptibility.

## Methods

### Literature search

A comprehensive literature search was conducted in PubMed, Embase, Web of Science, and the Cochrane Library of publications up to Apr 20th, 2022. The search terms used were as follows: (interferon-γ receptor OR interferon-γ receptor 1 OR IFNGR OR IFNGR1) AND tuberculosis. Further studies were identified from the reference lists, related articles and citation lists of each of papers identified from the initial searches.

The studies included in our meta-analysis met all the following criteria: (1) studies had to assess the association between IFNGR1 gene polymorphisms rs2234711, rs1327474, rs7749390, and TB susceptibility; (2) studies had a case-control or cohort design; (3) studies provided odds ratios (ORs) and the corresponding 95% confidence intervals (CIs) for at least for one model, or provided the allele frequencies to calculate the ORs and 95% CIs. Studies were excluded for the following criteria: (1) were reviews, meta-analysis, or case-reports; (2) provided no data about genotype distribution or allele frequency or ORs; (3) used duplicated data.

### Data extraction and quality assessment

The following data were extracted from the included studies: first author, publication year, country, ethnicity of participants, genotyping method, diagnosis assays for detection of TB, sample sizes in case groups and control groups, genotype distributions and allele frequency or the most fully adjusted ORs and 95% CIs. Two co-authors independently extracted the data from each study and any discrepancies were resolved *via* discussion with the third author.

The methodological quality of the included studies was assessed using the Newcastle-Ottawa Scale (NOS) (31). Studies with a NOS star of 0–3, 4–6, and 7–9 were considered as low, moderate, and high quality, respectively. Departure from the Hardy Weinberg equilibrium (HWE) for the control group in each study was assessed with Pearson's goodness-of-fit chi-square test with one degree of freedom or was extracted from the studies which did not provide genotype distribution.

### Statistical analysis

OR was used as a measure of the association between the IFNGR1 gene polymorphisms and TB susceptibility to combine the results. We used the most fully adjusted ORs and 95%CIs from each study for meta-analysis. When the adjusted ORs and 95%CIs were not provided by the included studies, we used the gene alle frequencies to compute the crude ORs and 95%CIs. The overall ORs and corresponding 95%CIs was pooled by the random-effect model. Heterogeneity across studies was detected by the Cochran Chi-square (significance level at *P* < 0.10) and Higgins I^2^ statistic (32, 33). The Begg's test (34) and the Egger's test (35) were used to assess publication bias. We pooled the ORs of associations between each SNP (for instance, the rs2234711) with TB susceptibility for six comparison models, including the dominant model (CC+CT vs. TT), allele model (C vs. T), homozygote model (CC vs. TT), heterozygote model (CT vs. TT), recessive model (CC vs. TT+CT), and additive model (CC vs. CT vs. TT). Sensitivity analysis was performed by individually removing the included studies to assess the stability of results. Subgroup analysis was conducted to determine whether there was an association between the IFNGR1 gene polymorphism and TB susceptibility in different ethnicities. All analyses were performed using STATA statistics software (Version 12.0, Stata Corporation, College Station, Texas 77, 845 United States). The statistical tests were all two-sided with a significance level of 0.05, unless otherwise specified.

## Results

### Literature search

**Figure 1:**
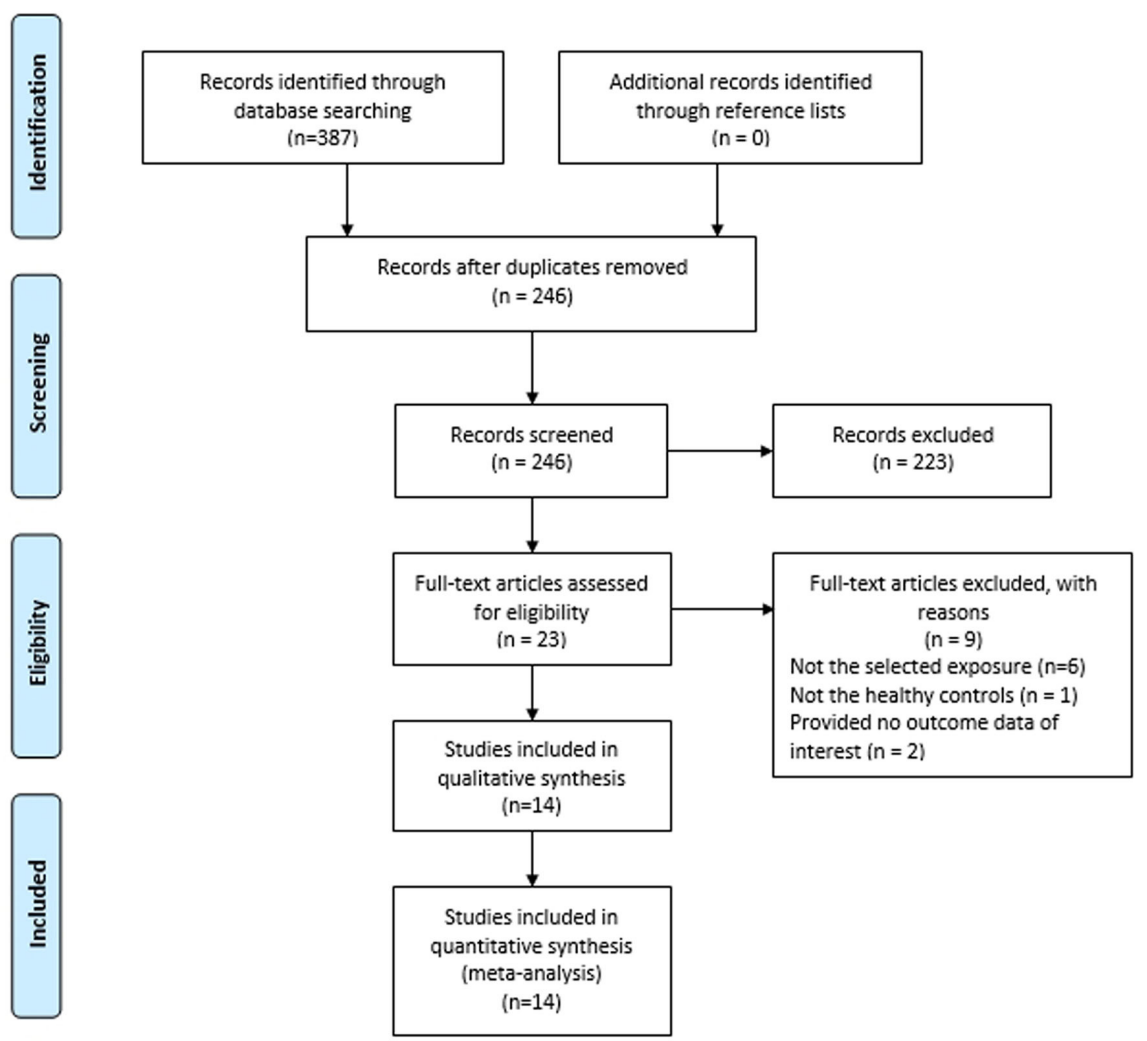
Flow diagram for identification of eligible studies for this meta-analysis.

Figure 1 presents the study selection flow. A total of 387 publications were retrieved from preliminary database search. Of these, 141 articles were removed as they were duplicates, leaving 246 articles for further review. By reading titles and abstracts, 223 were excluded as they were not relevant to our study. Through reading the full text of the remaining 23 articles, we further excluded 9 articles which did not meet the inclusion criteria. Finally, we included 14 articles with 15 case-control studies for the meta-analysis (17–30).

### Characteristics of included studies

The general characteristics of each included study are summarized in Table 1. In total, 15,988 participants were included across all studies with 7,699 TB cases and 8,289 controls. Eight articles (21–23, 26–30) were performed in Asians, four (17, 18, 20, 24) were performed in Africans, and two (19, 25) were conducted in Europeans. Eleven (17–25, 27, 28), eight (17, 19, 21, 22, 26, 27, 29, 30), and seven (17, 21, 23–25, 29, 30) studies reported the rs2234711, rs1327474, and rs7749390 polymorphisms, respectively. For rs2234711, the genotype distributions in the controls deviated from the HWE in two studies (18, 20); for rs1237474, the genotype distributions in the controls deviated from the HWE in one study (30); the genotype distributions in the controls were consistent with the HWE for rs7749390. The average NOS score for included studies was 7.4 (standard deviation: 1.1) (Supplemental Table 1).

**Table 1:** Basic information of studies included in this analysis.

**Study**	**Country, ethnicity**	**Sample size (case/control)**	**Diagnosed method**	**Genotyping method**	**Type**	**Specimen**	**SNPs**	**HWE**
Awomoyi et al. (17)	Gambia, African	320/320	Sputum smear	PCR-RFLP	PTB	Blood	rs1327474, rs2234711, rs7749390	>0.05
Bulat-Kardum (18)	Croatia, European	244/521	Sputum smear and Chest x-ray	PCR	TB	Blood	rs1327474, rs2234711	>0.05
Cooke et al. (19)	Gambia, Guinea Bissau, and the Republic of Conakry, African	682/619	Sputum smear or culture	PCR-ARMS	PTB	Blood	rs2234711	<0.05
Hwang et al. (20)	Korea, Asian	80/80	Sputum smear	PCR-ARMS	PTB	Blood	rs1327474, rs2234711	>0.05
He et al. (21)	China, Asian	222/188	Sputum smear or culture; and/or Chest x-ray	PCR	PTB or EPTB	Blood	rs1327474, rs2234711, rs7749390	>0.05
Lü et al. (22)	China, Asian	1,434/1,412	Sputum smear or culture; and/or Chest x-ray	PCR	PTB	Blood	rs2234711, rs7749390	>0.05
Rudko et al. (24)	Russians, European	238/265	NA	PCR-RFLP	Primary lung TB and severe secondary TB	Blood	rs2234711, rs7749390	>0.05
	Russians, European	331/279	NA	PCR-RFLP	Primary lung TB and severe secondary TB	Blood	rs2234711, rs7749390	>0.05
Naderi et al. (23)	Iran, Asian	173/163	Clinical symptoms, sputum smear and Chest x-ray	PCR	PTB	Blood	rs1327474, rs7749390	>0.05 (rs7749390), <0.05 (rs1327474)
Shin et al. (25)	Korean, Asian	673/592	Clinical symptom and culture	PCR	PTB	Blood	rs1327474, rs2234711	>0.05
Mayer (26)	Ghana, African	1,999/2,589	Sputum smear and/or culture	PCR-HRM	PTB	Blood	rs2234711, rs7749390	>0.05
Shamsi et al. (27)	Iran, Asian	100/100	Culture	PCR-RFLP	PTB	Blood	rs1327474	>0.05
He et al. (28)	China, Asian	467/503	Clinical symptoms, sputum smear and Chest x-ray	PCR	PTB	Blood	rs1327474, rs7749390	>0.05
Ali et al. (29)	Sudan, African	100/50	Sputum smear	PCR-RFLP	PTB	Sputum and blood samples	rs2234711	<0.05
Wu et al. (30)	China, Asian	636/608	Clinical symptoms, sputum smear and Chest x-ray	iMLDR	TB	Blood	rs2234711	>0.05

TB, tuberculosis; PTB, pulmonary tuberculosis; EPTB, extrapulmonary tuberculosis; PCR, polymerase chain reaction; PCR-RFLP, PCR-restriction fragment length polymorphism; PCR-ARMS, PCR-Amplification refractory mutation system; PCR-HRM, PCR-high-resolution melt; iMLDR, improved multiplex ligation detection reaction.

### Association of IFNGR1 rs2234711 polymorphism with TB susceptibility

**Figure 2:**
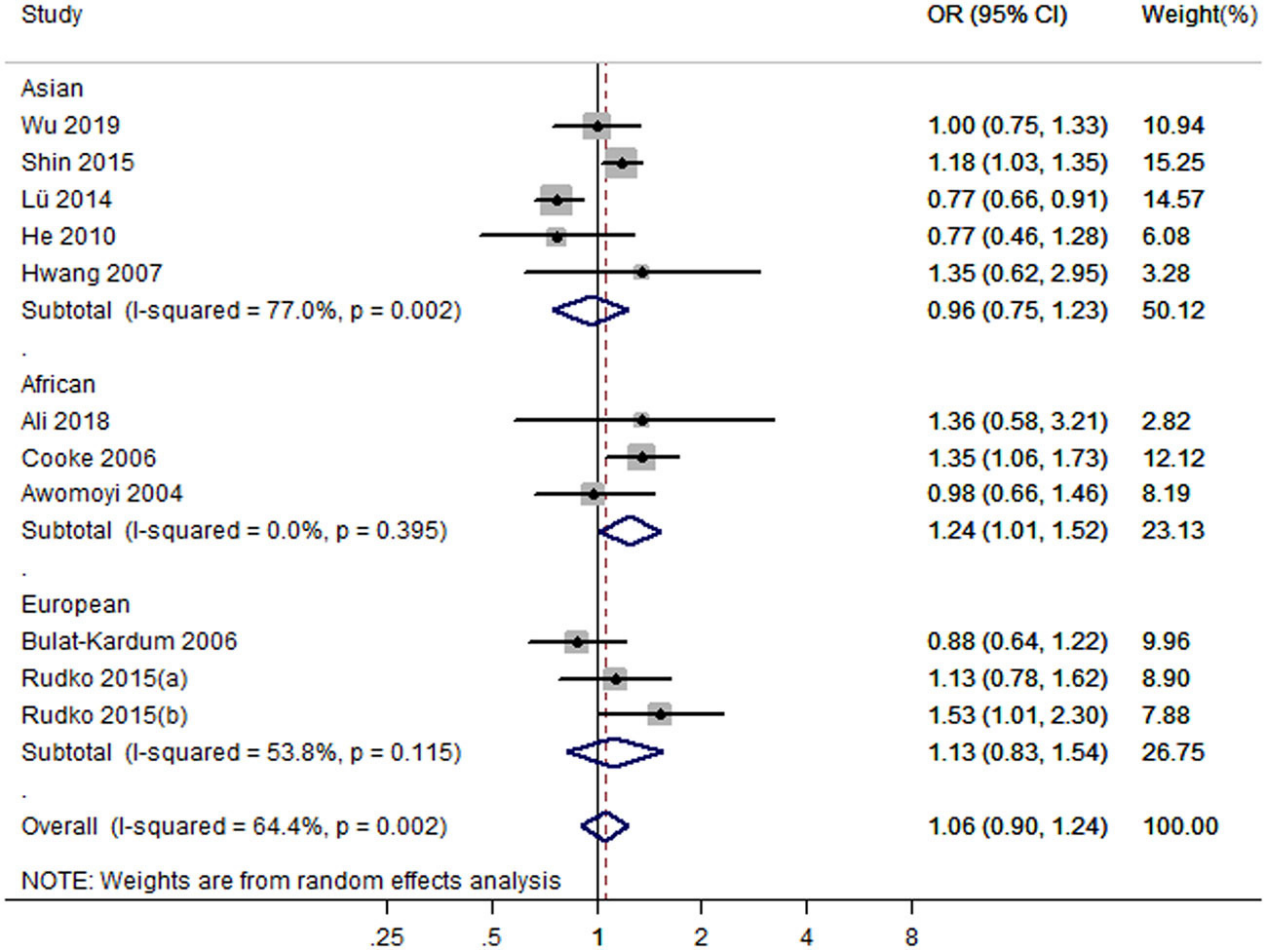
Forest plot of association between IFNGR1 rs2234711 polymorphism and tuberculosis susceptibility (dominant model: CC + CT vs. TT).

Eleven studies with 12 case-control studies (17–25, 27, 28) (6,989 cases and 7,523 controls) were included in the meta-analysis on the association between the IFNGR1 rs2234711 polymorphism and TB susceptibility. The pooled ORs indicated no significant association in dominant model (CC + CT vs. TT) (OR = 1.06, 95% CI 0.90–1.24) (Figure 2), allele model (C vs. T) (OR = 0.99, 95% CI 0.90–1.08), recessive model (CC vs. TT + CT) (OR = 1.02, 95% CI 0.94–1.11), heterozygote model (CT vs. TT) (OR = 1.05, 95% CI 0.87–1.26), homozygote model (CC vs. TT) (OR = 0.98, 95% CI 0.84–1.16), and additive model (CC vs. CT vs. TT) (OR = 1.01, 95% CI 0.91–1.11). The association of the IFNGR1 rs2234711 polymorphism with TB susceptibility was significant in Africans (dominant model: OR = 1.24, 95% CI: 1.01–1.52) and Asians (homozygote model: OR = 0.82, 95% CI: 0.70–0.98), respectively (Table 2).

**Table 2:** Results of association between IFNGR1 gene polymorphisms and TB susceptibility.

**Polymorphisms**	**Genetic models**	**Subgroup**	***n***	**OR (95%CI)**	**I^2^ (%)**	***P* for heterogeneity**
rs1327474	Dominant model					
		Total	8	0.83 (0.65, 1.05)	47.8	0.063
		Asian	6	0.78 (0.56, 1.09)	58.4	0.034
		African	1	0.83 (0.44, 1.55)	NA	NA
		European	1	1.01 (0.73, 1.40)	NA	NA
	Allele model					
		Total	7	0.81 (0.61, 1.08)	66.2	0.007
		Asian	5	0.74 (0.48, 1.15)	73.4	0.005
		African	1	0.84 (0.46, 1.53)	NA	NA
		European	1	1.03 (0.83, 1.28)	NA	NA
	Recessive model					
		Total	7	1.02 (0.73, 1.43)	0	0.473
		Asian	5	0.79 (0.34, 1.83)	22.9	0.266
		African	1	0.97 (0.06, 15.60)	NA	NA
		European	1	1.09 (0.74, 1.61)	NA	NA
	Heterozygote model					
		Total	7	0.81 (0.62, 1.07)	42.9	0.105
		Asian	5	0.76 (0.51, 1.14)	57	0.054
		African	1	0.82 (0.44, 1.54)	NA	NA
		European	1	0.98 (0.70, 1.38)	NA	NA
	Homozygote model					
		Total	6	0.92 (0.52, 1.63)	24.8	0.248
		Asian	4	0.72 (0.23, 2.31)	51.9	0.1
		African	1	0.95 (0.06, 15.18)	NA	NA
		European	1	1.08 (0.69, 1.69)	NA	NA
	Additive model					
		Total	6	0.83 (0.61, 1.12)	68.5	0.007
		Asian	4	0.76 (0.47, 1.22)	77.1	0.004
		African	1	0.85 (0.47, 1.53)	NA	NA
		European	1	1.03 (0.83, 1.28)	NA	NA
rs2234711	Dominant model					
		Total	11	1.06 (0.90, 1.24)	64.4	0.002
		Asian	5	0.96 (0.75, 1.23)	77	0.002
		African	3	1.24 (1.01, 1.52)	0	0.395
		European	3	1.13 (0.83, 1.54)	53.8	0.115
	Allele model					
		Total	11	0.99 (0.90, 1.08)	59.7	0.006
		Asian	4	0.89 (0.79, 0.99)	31	0.226
		African	4	1.03 (0.87, 1.23)	66.8	0.029
		European	3	1.06 (0.89, 1.26)	27.3	0.253
	Recessive model					
		Total	11	1.02 (0.94, 1.11)	0	0.53
		Asian	5	0.99 (0.86, 1.13)	35.4	0.185
		African	3	1.10 (0.89, 1.36)	0	0.868
		European	3	1.00 (0.76, 1.31)	0	0.37
	Heterozygote model					
		Total	10	1.05 (0.87, 1.26)	58.2	0.011
		Asian	4	0.88 (0.71, 1.10)	35.6	0.198
		African	3	1.24 (1.00, 1.54)	0	0.447
		European	3	1.15 (0.79, 1.65)	62.9	0.068
	Homozygote model					
		Total	10	0.98 (0.84, 1.16)	25.6	0.208
		Asian	4	0.82 (0.70, 0.98)	0	0.556
		African	3	1.26 (0.98, 1.62)	0	0.549
		European	3	1.04 (0.7, 1.40)	0	0.607
	Additive model					
		Total	10	1.01 (0.92, 1.11)	45	0.06
		Asian	4	0.90 (0.83, 0.97)	0	0.511
		African	3	1.13 (0.99, 1.28)	0	0.489
		European	3	1.06 (0.89, 1.25)	23.2	0.272
rs7749390	Dominant model					
		Total	7	1.02 (0.81, 1.27)	61.9	0.015
		Asian	4	1.00 (0.71, 1.41)	78.1	0.003
		African	1	0.96 (0.65, 1.42)	NA	NA
		European	2	1.15 (0.79, 1.67)	0	0.361
	Allele model					
		Total	8	0.98 (0.88, 1.09)	58.5	0.018
		Asian	4	1.07 (0.88, 1.29)	72.4	0.012
		African	2	0.89 (0.82, 0.98)	0	0.481
		European	2	0.89 (0.66, 1.20)	55.8	0.132
	Recessive model					
		Total	7	1.02 (0.85, 1.23)	47.2	0.078
		Asian	4	1.16 (0.92, 1.47)	47.9	0.124
		African	1	0.99 (0.68, 1.45)	NA	NA
		European	2	0.77 (0.57, 1.05)	19.5	0.265
	Heterozygote model					
		Total	7	1.00 (0.78, 1.27)	62.5	0.014
		Asian	4	0.93 (0.66, 1.31)	76.1	0.006
		African	1	0.96 (0.63, 1.47)	NA	NA
		European	2	1.30 (0.88, 1.27)	0	0.64
	Homozygote model					
		Total	7	1.05 (0.83, 1.34)	50.8	0.058
		Asian	4	1.14 (0.77, 1.69)	73.2	0.011
		African	1	0.97 (0.60, 1.56)	NA	NA
		European	2	0.97 (0.64, 1.46)	0	0.324
	Additive model					
		Total	7	1.03 (0.90, 1.19)	64.5	0.01
		Asian	4	1.07 (0.87, 1.30)	75.1	0.007
		African	1	1.21 (0.92, 1.60)	NA	NA
		European	2	0.89 (0.67, 1.19)	52.9	0.145

### Association of IFNGR1 rs1327474 polymorphism with TB susceptibility

**Figure 3:**
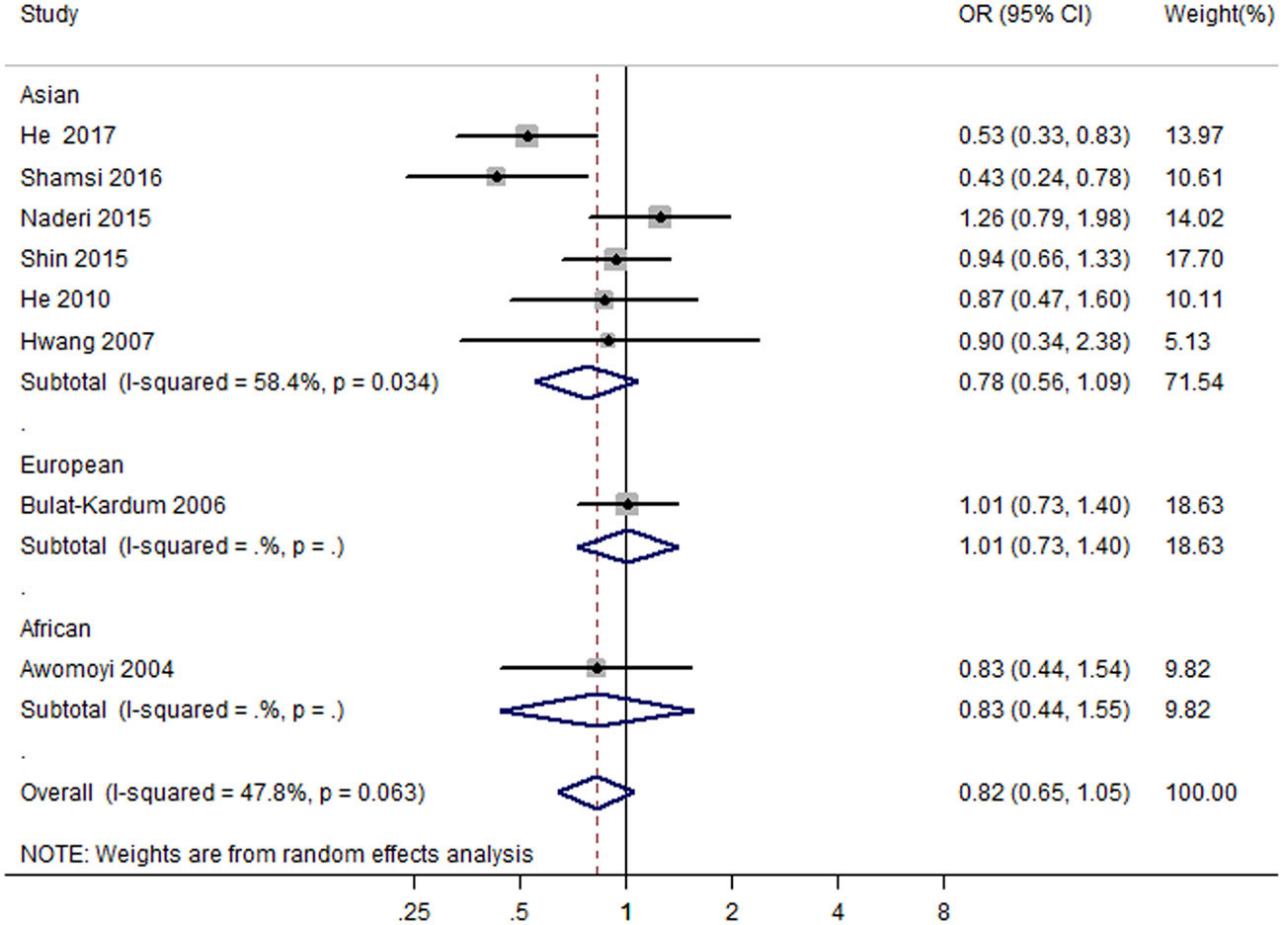
Forest plot of association between IFNGR1 rs1327474 polymorphism and tuberculosis susceptibility (dominant model: GG + AG vs. AA).

Eight case-control studies (17, 19, 21, 22, 26, 27, 29, 30) (2,279 cases and 2,467 controls) were included in the meta-analysis on the association between IFNGR1 rs1327474 polymorphism and TB susceptibility. The association of IFNGR1 rs1327474 polymorphism with TB susceptibility was not detected in the dominant model (GG + AG vs. AA) (OR = 0.83, 95% CI: 0.65–1.05) (Figure 3), allele model (G vs. A) (OR = 0.81, 95% CI 0.61–1.08), recessive model (GG vs. AG + AA) (OR = 1.02, 95% CI 0.73–1.43), heterozygote model (AG vs. AA) (OR = 0.81, 95% CI 0.62–1.07), homozygote model (GG vs. AA) (OR = 0.92, 95% CI 0.52–1.63), and additive model (GG vs. GA vs. AA) (OR = 0.83, 95% CI 0.61–1.12) (Table 2).

### Association of IFNGR1 rs7749390 polymorphism with TB susceptibility

**Figure 4:**
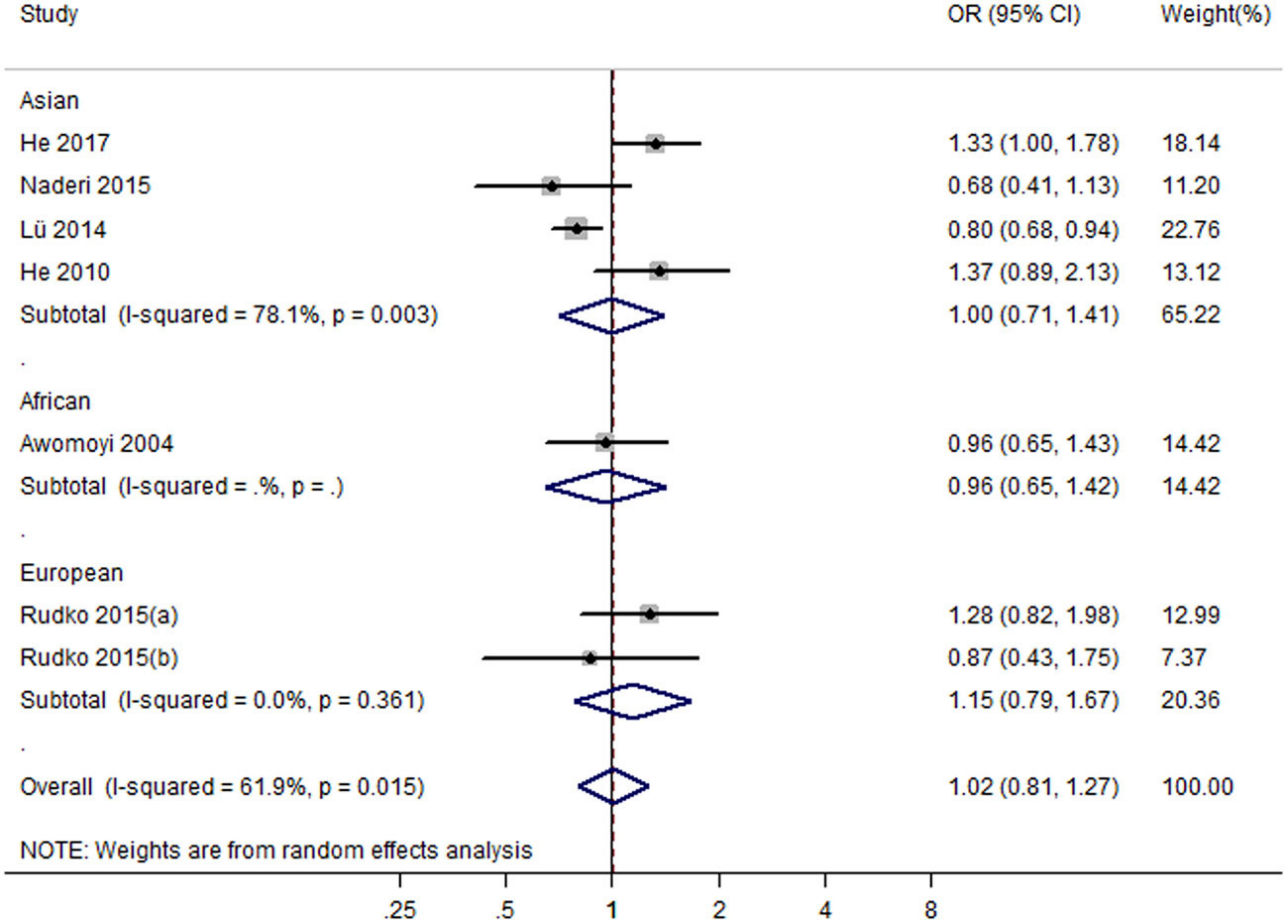
Forest plot of association between IFNGR1 rs7749390 polymorphism and tuberculosis susceptibility (dominant model: TT + TC vs. CC).

Seven studies with eight case-control studies (17, 21, 23–25, 29, 30) (5,184 cases and 5,719 controls) were included in the meta-analysis on the association between the IFNGR1 rs7749390 polymorphism and the TB susceptibility. The pooled ORs from the studies indicated no significant association in the dominant model (TT + TC vs. CC) (OR = 1.02, 95% CI 0.81–1.27) (Figure 4), allele model (T vs. C) (OR = 0.98, 95% CI 0.88–1.09), recessive model (TT vs. CC + TC) (OR = 1.02, 95% CI 0.85–1.23), heterozygote model (TC vs. CC) (OR = 1.00, 95% CI 0.78–1.27), homozygote model (TT vs. CC) (OR = 1.05, 95% CI 0.83–1.34), and additive model (TT vs. TC vs CC) (OR = 1.03, 95% CI 0.90–1.19). However, the association between the IFNGR1 rs7749390 polymorphism and TB susceptibility in the allele model was significant in Africans (OR = 0.89, 95% CI: 0.82–0.98) (Table 2).

### Sensitivity analysis

In the sensitivity analysis of the results from the dominant models, the influence of each individual data set on the pooled OR was assessed by removing each study individually. When excluding the results from Lu et al.'s (23) study, the pooled OR (1.13, 95% CI: 1.02–1.26) indicated an association between rs2234711 and TB susceptibility. When excluding the result from Naderi et al.'s study (30), rs1327474 was suggested to have a protective effect on TB susceptibility (OR = 0.77, 95% CI: 0.60, 0.99). For rs7749390, the sensitivity analysis demonstrated relatively robust results with an OR range from 0.95(95% CI: 0.76, 1.18) when excluding He et al.'s (21) study to 1.10 (95% CI: 0.89, 1.37) when omitting Lu et al's. (23) study.

To assess the possible effects of studies in which SNPs were not in HWE, we conducted a sensitivity analysis by omitting these studies and re-pooled the results. However, the overall ORs did not alter markedly in each model, except that rs1327474 seemed to be a protective polymorphism for TB risk in dominant model (OR = 0.77, 95% CI: 0.60–0.99).

### Publication bias

The Begg's test and Egger's test were used to evaluate the publication bias of the included studies in this meta-analysis. The shape of Begg's funnel plot revealed no evidence of obvious asymmetry in all genetic models, which showed no potential publication bias (Supplementary Figures 1–3). Meanwhile, the statistical results from Egger's test and Begg's test also indicating that there was no publication bias across studies (all *p* > 0.05).

## Discussion

To our knowledge, this is the first meta-analysis that systematically assessed the association of the IFNGR1 rs2234711, rs1327474, and rs7749390 polymorphisms with TB susceptibility. Based on 14 case-control studies with 15,988 participants, we found that IFNGR1 gene polymorphism was associated with TB susceptibility in some ethnic groups.

Various gene polymorphisms in the IFNGR1 are suggested to be associated with susceptibility of diverse diseases, such as inflammatory and virus-associated disorders (36–38). For the association of IFNGR1 gene polymorphisms with TB susceptibility, Wang et al. (39) conducted a meta-analysis focused on IFNGR1 rs2234711 polymorphism. Based on a total of six studies comprising 1,497 confirmed TB cases and 1,802 controls, Wang et al. (39) observed no significant association between IFNGR1 rs2234711 polymorphism and TB susceptibility. However, like the present meta-analysis, the association between IFNGR1 rs2234711 polymorphism and TB susceptibility was also found in Africans (dominant model: CC + TC vs. TT, OR = 1.24, 95% CI 1.02–1.51) in Wang et al.'s meta-analysis. Nevertheless, the results from the present meta-analysis were more reliable because of more included studies and larger sample size.

The rs2234711 polymorphism is located within the AP4 binding site in the 5' upstream region of the gene. This region has been associated with susceptibility to some infectious diseases, including TB (36, 37, 40). In this meta-analysis, significant association of rs2234711 polymorphism with TB susceptibility was detected in Africans (dominant model) and in Asians (homozygote model, allele model, and additive model). The result indicated that the c allele might be a potential risk factor for TB infection in Africans, whereas providing a protective effect against TB infection in Asians. Therefore, rs2234711 polymorphism may exert inversed effect on Africans and Asians. The rs7749390 polymorphism is located in an exon/intron splice site and likely to influence the intron–exon splicing process. Significant association were detected in the allele model among Africans in our study, which indicated that the T allele was associated with decreased risk for TB in the African population. The rs1327474 polymorphism, which is located in the promoter region of the IFNGR1 gene, has been shown to have higher transcriptional activity (21). He et al. reported that the rs1327474 variant was associated with a decreased risk of pulmonary TB (21). In this meta-analysis, however, similar association was only found in Africans (dominant model).

Gene polymorphisms are complicated and fluctuating, especially among different ethnicities and populations from different regions (41–43). Studies have revealed that discrepancy in distribution of IFNGR1 genotype may exist in different populations (44), which may contribute to the inconsistent associations of genetic polymorphisms with TB susceptibility. The minor allele frequency of the SNP s2234711 varies greatly in different areas, ranging from 0% in European populations to 60% in African–American populations (23). Furthermore, studies have shown that the genetic variation of the bacteria and the phylogeographic distribution of the TB-causing bacteria differed from ethnicity or populations, and patients of different ethnicity presents different TB phenotypes (45). Moreover, the burden of TB is much higher in Asia and Africa geographically than that in other regions (46–48). There is a necessity to further explore the possible effects of IFNGR1 gene polymorphisms on TB risk among more specifically defined ethnicities, such as the Caucasians, Africans, and Mongolians.

This meta-analysis indicated that Africans and Asians seem to be more sensitive to IFNGR1 gene polymorphisms compared with other ethnicities. It might be partially explained by the several factors. First, most of the original studies on TB were conducted in Asian and African populations, which provides more statistical power for detecting significant results. Second, the prevalence of TB was relatively high among Asians or Africans within the studies assessed here (49, 50), therefore more gene mutations could be detected. Third, environmental factors, such as diet, rainfall capacity, and social-economic factors in Asians or Africans may facilitate TB infection.

The present study systematically assessed the association between IFNGR1 gene polymorphisms and TB susceptibility. Strength of this meta-analysis was that the overall results were generally robust, as suggested by the subgroup analysis and sensitivity analysis. Nevertheless, some limitations should be noted. First, all included studies were case-control studies. Some included studies used matched controls (e.g., age and sex matched), while others did not. Confounding factors, such as, age, sex, HIV status, and TB severity, were not adjusted, which may lead to underestimation or overestimation of the risk estimates. Second, the number of included studies were relatively limited, and there was significant heterogeneity across studies, the geographic region, characteristics of participants, diagnosed method, genotyping method may all contribute to the heterogeneity across studies, the strength of our findings may be weakened. Third, this meta-analysis included studies (18, 20, 30) in which the SNPs were not in HWE, stability of the results could be affected, although sensitivity analysis omitting these studies (18, 20, 30) showed that the re-pooled ORs were consistent with the overall ORs.

In conclusion, this meta-analysis indicated that IFNGR1 rs2234711 polymorphism was associated with increased TB susceptibility in Africans and decreased TB susceptibility in Asians, while IFNGR1 rs7749390 polymorphism was associated with decreased TB susceptibility in Africans. This study provided evidence that IFNGR1 rs2234711 might be involved in the pathogenesis of TB in certain ethnic groups. However, further studies with larger sample size and more stringent design are warranted to confirm the present findings among different ethnic groups.

## Data availability statement

The original contributions presented in the study are included in the article/Supplementary material, further inquiries can be directed to the corresponding authors.

## Author contributions

XY and JC conceived and designed the experiments. LC, FZ, and YW performed the experiments. LC, JC, and FZ analyzed the data. LC, XY, and YW contributed reagents, materials, and analysis tools. LC and FZ wrote the paper. All authors reviewed the manuscript. All authors contributed to the article and approved the submitted version.

## Conflict of interest

The authors declare that the research was conducted in the absence of any commercial or financial relationships that could be construed as a potential conflict of interest.

## Publisher's note

All claims expressed in this article are solely those of the authors and do not necessarily represent those of their affiliated organizations, or those of the publisher, the editors and the reviewers. Any product that may be evaluated in this article, or claim that may be made by its manufacturer, is not guaranteed or endorsed by the publisher.
