# Supplementary material for: Association between IFNGR1 gene polymorphisms and tuberculosis susceptibility: A meta-analysis
Source: Front Public Health. 2022 Sep 6;10:976221. doi: 10.3389/fpubh.2022.976221 (PMC9485675; doi:10.3389/fpubh.2022.976221)
Supplement: Supplementary file 1 [file Data_Sheet_1.pdf]

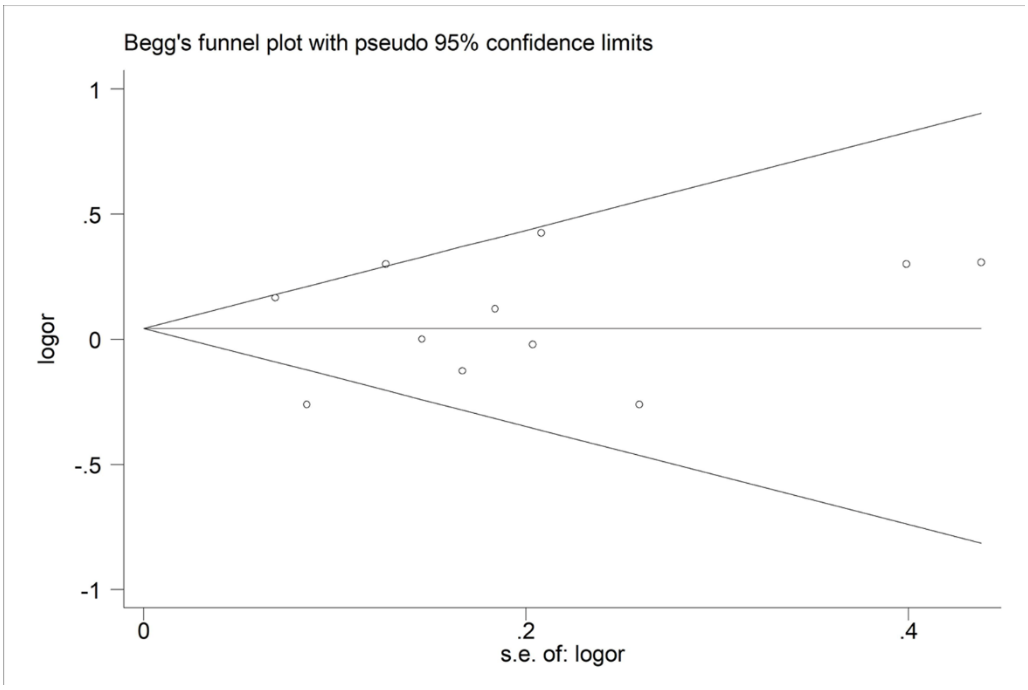

Supplemental Figure 1. Begg's funnel plot of IFNGR1 rs2234711 polymorphism on Tuberculosis risk (dominant model: CC+CT vs. TT).

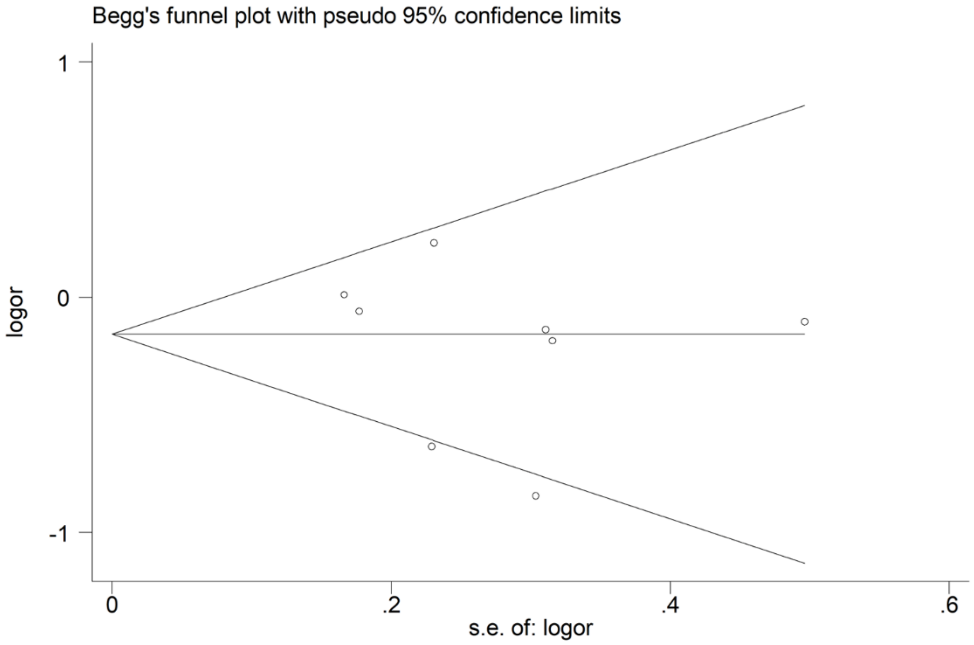

Supplemental Figure 2. Begg's funnel plot of IFNGR1 rs1327474 polymorphism on Tuberculosis risk (dominant model: GG+AG vs AA).

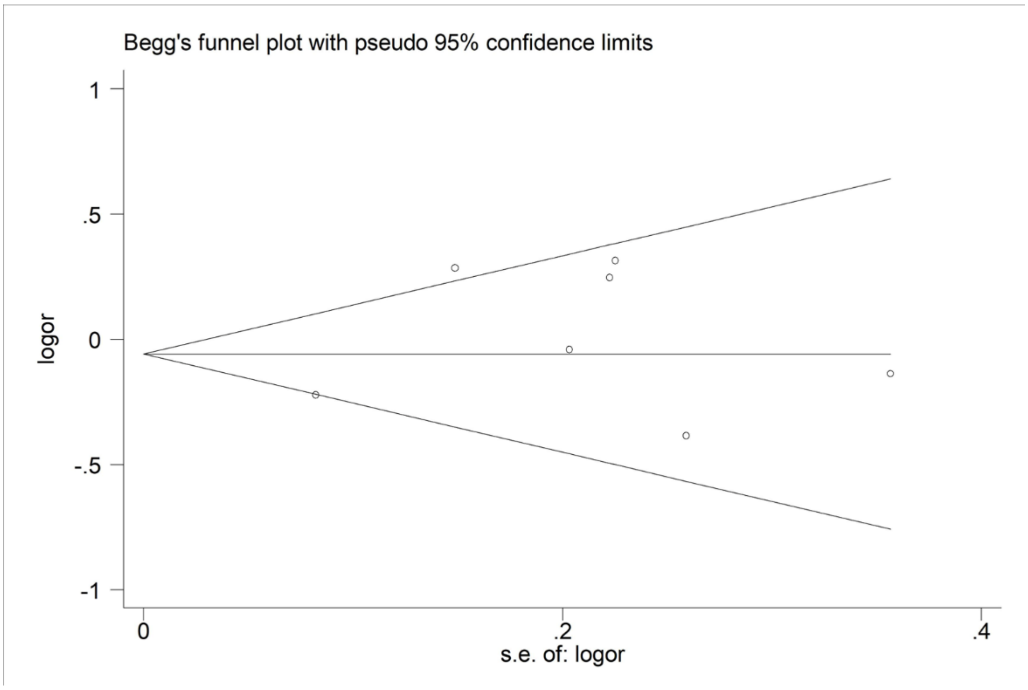

Supplemental Figure 3. Begg’s funnel plot of IFNGR1 rs7749390 polymorphism on Tuberculosis risk (dominant model: TT+TC vs. CC).
