# Supplementary material for: Association between IFNGR1 gene polymorphisms and tuberculosis susceptibility: A meta-analysis
Source: Front Public Health. 2022 Sep 6;10:976221. doi: 10.3389/fpubh.2022.976221 (PMC9485675; doi:10.3389/fpubh.2022.976221)
Supplement: Supplementary file 2 [file Table_1.DOC]

**Supplemental Table 1.** Methodological quality of included case-control studies

| First author | Adequate case definition | Representativeness of the cases | Selection of Controls | Definition of Controls | Comparability of cases and controls ^1^ | Ascertainment of exposure | Same method of ascertainment for cases and controls | Non-Response rate | Total quality scores |
| --- | --- | --- | --- | --- | --- | --- | --- | --- | --- |
| Wu 2019 | ☆ | ☆ | — | ☆ | ☆☆ | ☆ | ☆ | ☆ | 8 |
| Ali 2018 | ☆ | ☆ | ☆ | ☆ | — | ☆ | ☆ | ☆ | 7 |
| SHIN 2015 | ☆ | ☆ | ☆ | ☆ | — | ☆ | ☆ | ☆ | 7 |
| Lü 2014 | ☆ | ☆ | ☆ | ☆ | ☆☆ | ☆ | ☆ | ☆ | 9 |
| He 2010 | ☆ | ☆ | — | ☆ | ☆ | ☆ | ☆ | ☆ | 7 |
| Hwang 2007 | ☆ | ☆ | ☆ | ☆ | — | ☆ | ☆ | ☆ | 7 |
| Cooke 2006 | ☆ | ☆ | — | ☆ | ☆☆ | ☆ | ☆ | ☆ | 8 |
| Bulat-Kardum 2005 | ☆ | ☆ | — | ☆ | ☆ | ☆ | ☆ | ☆ | 7 |
| Awomoyi 2004 | ☆ | ☆ | — | ☆ | ☆☆ | ☆ | ☆ | ☆ | 8 |
| Rudko 2015(a)^2^ | — | ☆ | — | ☆ | — | ☆ | ☆ | ☆ | 5 |
| Rudko 2015(b) | — | ☆ | — | ☆ | ☆ | ☆ | ☆ | ☆ | 7 |
| Mayer 2016 | ☆ | ☆ | ☆ | ☆ | ☆☆ | ☆ | ☆ | ☆ | 9 |

^1^ A maximum of 2 stars were assigned to this item. ^2^ Case-control study conducted in Russians from Tomsk region.
